# Was machen Urologen in der Praxis? Ein erster „ungefilterter“ Blick auf die Versorgung

**DOI:** 10.1007/s00120-021-01545-1

**Published:** 2021-05-28

**Authors:** Peter J. Goebell, Sulafah El-Khadra, Marcus Horstmann, Peter Kollenbach, Gerson Lüdecke, Thomas Quack, Michael Rug, Michael Stephan-Odenthal, Kerstin Bode-Greuel, Saskia Zink, Patrick Lieberkühn, Matthias Schulze

**Affiliations:** 1 Urologische und Kinderurologische Universitätsklinik, Friedrich-Alexander-Universität Erlangen-Nürnberg, Krankenhausstraße 12, 91054 Erlangen, Deutschland; 2 Urologie am Kaiserdamm, Berlin, Deutschland; 3 HELIOS Krankenhaus St. Josefshospital Krefeld, Krefeld, Deutschland; 4 Urologie am Weinberg, Kassel, Deutschland; 5 Urologische Klinik, Uniklinikum Gießen und Marburg Standort Gießen, Gießen, Deutschland; 6 Urologische Praxis Dr. Quack, Plön, Deutschland; 7 Urologische Praxis Dr. Rug, Karlsruhe, Deutschland; 8 Gemeinschaftspraxis UROLOGIE RHEIN.BERG, Leverkusen, Deutschland; 9 Deutsches Institut für Fachärztliche Versorgungsforschung GmbH (DIFA), Berlin, Deutschland; 10 Speziallabor und Lasermedizin, Praxis für Urologie, Andrologie, Onkologie, Markkleeberg, Deutschland

**Keywords:** Versorgung, Versorgungsforschung, UROgister, UROscience, Berufsverband der Deutschen Urologen (BvDU), Healthcare science, Real-world data, UROgister, UROscience, Berufsverband der Deutschen Urologen (BvDU)

## Abstract

Auch (berufs)politisch hat das Thema Versorgung und Versorgungsforschung an Relevanz gewonnen und stellt für die Gesundheitspolitik und die an der Versorgung Beteiligten eine wichtige Basis dar. Der Zugang zu relevanten Daten und die Möglichkeiten zur Analyse sind daher umkämpfter denn je, liefern sie doch die besten Argumente und Fakten in jedem Diskurs um letztlich begrenzte Ressourcen des gesamten Gesundheitssektors. Allen randomisierten klinischen Studien und prospektiven Datensammlungen ist gemein, dass sie einen festen vordefinierten Rahmen der zu erfassenden verschiedenen Datenpunkte zugrunde gelegt haben, um störende Einflussfaktoren möglichst zu kontrollieren. Auch Analysen aus retrospektiven Datensammlungen benutzen eine vordefinierte Auswertematrix und filtern die vorhandenen Daten auf diese festgelegten Datenpunkte. Ein ungefilterter Blick auf alle Daten wäre aber der Idealzustand. Näherungsweise sollten in diesem Projekt so viele Daten wir möglich „ungefiltert“ erfasst werden und in einem Datenpool gesammelt werden, die dann durch sich ständig verbessernde Analysealgorithmen aufgearbeitet werden können. Über die automatisierte Extraktion von Daten aus dem Arzt‑/Praxisinformationssystem (AIS) in UROscience wird ein Datenpool entstehen, der für viele Fragestellungen zur Versorgungsrealität genutzt werden kann. Die hier vorgestellte erste Analyse zeigt, dass auf Basis der bereits vorhandenen Daten eine vielseitig einsetzbare Stichprobe für die Darstellung des Versorgungsalltags aus der urologischen Praxis zur Verfügung steht.

## Abstract (en)

The topic of routine medical care data and healthcare science has gained in relevance and provides an important basis for both healthcare policymakers and those providing care. Access to relevant data and the ability to analyze these is highly competitive as it yields the most compelling arguments and strong facts in any discourse on the ultimately limited resources of the entire healthcare sector. All randomized clinical trials and prospective data collections harbor the inherent similarity that they contain data within a predefined frame of data elements in order to control for any confounding factors. In addition, analyses using retrospective data collections use a predefined evaluation matrix and filter the existing data according to these established data elements. However, an unfiltered and un(pre)specified view to all data would be ideal. An approximation to this goal as part of this project could be the unfiltered collection of as much data as possible and their collection in a data pool, which then could be processed, in a constantly improving analyses algorithm. The automated self-extraction of data from the private-practice information technology (IT) system to UROscience will create a data pool which could be used to answer many different questions related to the reality of healthcare. The preliminary analyses presented here demonstrate that, on basis of the existing data, this versatile sample is available to provide insight into the treatment reality of urologic outpatient care.

## Einleitung

Versorgung und Versorgungsforschung stehen auch in der Urologie immer wieder im Fokus der wissenschaftlichen und allgemeinen Diskussionen. Es hat in der Vergangenheit hierzu einige Positionspapiere und viele interessante Beiträge auf den verschiedensten Plattformen gegeben und es gibt zahlreiche spannende Projekte aus der Urologie, die entweder retrospektiv oder prospektiv beachtliche Datensammlungen generiert haben und weitere, die Daten generieren werden [1–11]. (Fast) allen diesen Projekten ist gemein, dass sie einen festen vordefinierten Rahmen der zu erfassenden verschiedenen Datenpunkte zugrunde gelegt haben. Das hat den Vorteil einer hohen Homogenität und „Analysefähigkeit“ der Daten, was die Verlässlichkeit der generierten Aussagen zu erhöhen scheint. Auch benötigt man eine kleinere Stichprobe, da Unterschiede klarer erkennbar werden, wenn sie nicht durch ein „Grundrauschen“ ungefilterter Daten überdeckt werden.

Diese Form der Analytik hat aber auch – im Vergleich zu „Big Data“ – einige Limitationen und Nachteile: Der Blick auf die Grundgesamtheit erfolgt immer durch den vorher definierten Filter, der durch die Festlegung der Datenpunkte entstanden ist, die Eingabe der Daten ist mit einem nicht unerheblichen Zeit‑, Personal- und Kostenaufwand verbunden und es können nur die Korrelationen aufgedeckt werden, die zwischen den (vor)definierten Datenpunkten bestehen.

Ein „ungefilterter“ Blick auf „alle“ Daten wäre ideal – ist aber unrealistisch. Eine Näherung könnte aber darin bestehen, dass man ohne Vorauswahl und möglichst vollständig alle vorhandenen umfangreichen Daten in einen Pool einschleust. Da Zusammenhänge oder Beziehungen von Datenpunkten im Gesamtkollektiv nicht zugrunde gelegt werden, kann eine solche heterogene Grundgesamtheit besser Analyseverfahren wie z. B. Clusteranalysen unterzogen werden, als das bei prädefinierten Datensammlungen der Fall ist. Bei Analyseverfahren wie der Clusteranalyse geht es letztlich darum, mit einem Segmentierungsverfahren aus einer Grundgesamtheit homogene Teilmengen zu bilden, die nach gleichzeitiger Heranziehung „aller“ Eigenschaften zur Gruppenbildung gefunden werden können [12]. Hierbei können auch bisher unbekannte Beziehungen von Eigenschaften aufgedeckt werden um so zumindest hypothesengenerierend wertvolle Grundlagen für weitere Überprüfungen darzustellen. Auch andere Analyseverfahren (z. B. Netzwerkanalysen [13]), die v. a. aus der Soziologie entlehnt werden, zielen darauf ab, die Beziehungen von Datenpunkten zu charakterisieren und in einen „neuen“ Gesamtzusammenhang zu stellen. Auch hierbei können die verschiedenen Algorithmen lernend alternierend angepasst werden, um weitere Korrelationen aufdecken zu können. Diese „neuen“ Zusammenhänge können dann wiederum an definierten Datensets geprüft und an weiteren Kohorten prospektiv validiert werden. Erfolgreich ist die Anwendung und Transferierung von Netzwerkanalysen beispielsweise für die Erforschung verschiedener Markersignaturen bei Tumorpatienten bereits eingesetzt worden [14].

Ein weiterer extremer Vorteil des hier besprochenen Modells von UROscience besteht darin, Daten automatisiert aus dem Arzt‑/Praxisinformationssystem (AIS) zu extrahieren, was die Bereitstellung erleichtert und v. a. keinen zusätzlichen Arbeitsaufwand bedeutet. Ein Nachteil mag darin bestehen, dass eine Verbesserung der dann anonymisierten Daten nur über Umwege erreichbar ist, da eine „source-data verification“ nicht wie bei einer klassischen Datensammlung stattfinden kann.

**Figure 1:**
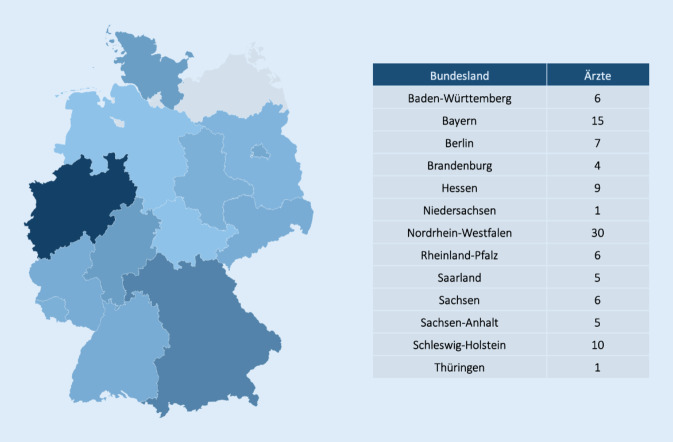

Als Pioniere innerhalb des Spitzenverbands Fachärzte Deutschlands e. V. (SpiFa) haben die urologischen Fachärzte aus dem Berufsverband der Deutschen Urologen (BvDU) gemeinsam mit dem DIFA (Deutsches Institut für Fachärztliche Versorgungsforschung) das Projekt UROgister/UROscience initiiert [15], dem zwei unterschiedliche Ansätze zugrunde liegen – der Aufbau einer urologischen Versorgungsforschungsdatenbank (UROscience) und die Entwicklung eines Tools zur Unterstützung der verpflichtenden Krebsregistermeldung (UROgister; https://difa-vf.de/urogister-krebsregistermeldung). Derzeit stellen 105 Urologen Daten für die Bewertung und Beforschung insbesondere berufspolitisch relevanter Fragestellungen aller Art zur Verfügung, die über ganz Deutschland verteilt sind (Abb. 1). Der dafür aus dem Hauptausschuss des BvDU ins Leben gerufene UROscience-Beirat unter dem Vorsitz von Prof. Peter J. Goebell begleitet den Umgang mit den urologischen Daten, bewertet und prüft Anfragen an die DIFA zu Analysen und kann Fragen aus der Urologie an die Datenbank aktiv platzieren, die besonders im berufspolitischen Kontext relevant erscheinen.

### Konkurrenz oder Ergänzung?

Immer wieder wurde in der Vergangenheit die Initiative als „Konkurrenzprodukt des Berufsverbands“ zu anderen Datensammlungen – v. a. dem d‑uo – angesehen [16], obwohl sich dieses Projekt deutlich von bisherigen Konzepten unterscheidet: Es greift „alle“ Daten aus dem AIS „ungefiltert“ ab, beschränkt sich nicht auf vordefinierte „Kohorten“ mit den entsprechenden prädeterminierten Datenpunkten, hat klare Limitationen im Hinblick auf die Rückverfolgung, ermöglicht andererseits Big-Data-Analytik und kann auch longitudinale Einflussfaktoren sichtbar machen, die zumindest hypothesengenerierend extrem wertvoll wären. Darüber hinaus dienen die generierten Zahlen auch der Abbildung der Versorgungsrealität und können helfen aufzuzeigen, wie die Versorgung in der Praxis funktioniert, was in seiner berufspolitischen Dimension wichtige Basis für den Dialog mit Kostenträgern und Politik sein kann, wenn hier aus „eigener Hand“ Daten und Fakten vorgelegt werden können [17]. Die Vorstellung, UROscience stelle eine Konkurrenz zu aktuell laufenden Projekten, wie etwa das d‑uo dar, muss vor diesem Hintergrund dringend revidiert werden – allenfalls stellt es eine wichtige zusätzliche Quelle zur Betrachtung der Versorgungsrealität dar und könnte auch bei möglichen gemeinsamen Projekten wichtige zusätzliche Aspekte liefern.

## Struktur der Datensammlung in UROscience

Die Erhebung von Daten aus der fachärztlichen urologischen Versorgung ist ein erster Schritt in Richtung eigener fächerübergreifender Versorgungsforschung der deutschen Fachärzteschaft. Geleitet wird diese Initiative von der Erkenntnis, dass sowohl in der berufspolitischen Positionierung als auch zu Forschungszwecken die aus der Versorgung stammenden Daten und daraus basierenden Erkenntnisse aus der täglichen Arbeit der Fachärzte mit ihren Patienten von fundamentaler Wichtigkeit sind. Die vom DIFA geleitete und koordinierte *Versorgungsforschung von Fachärzten für Fachärzte* wird fächerübergreifend über die im SpiFa vertretenen Facharztgruppen und auch darüber hinaus, derzeit von den Urologen ausgehend, schrittweise erweitert und ausgebaut werden. Die in DIFA *Science* zusammengefasste Datensammlung schafft die Möglichkeit für vertiefende Analysen zu Wirkungszusammenhängen sowie zum Erkennen und Beheben spezifischer Versorgungsprobleme. Die Fachärzte handeln hier im eigenen Interesse, um unabhängig und auf Augenhöhe auch mit den Körperschaften und Kostenträgern datengestützt argumentationsfähig zu werden. Überdies geben die Daten Auskunft zum Leistungsgeschehen in der eigenen Praxis. Verschreibungsverhalten, Verhältnis der Abrechnungen nach GOÄ und EBM sowie Vergleichswerte aus der eigenen Fachgruppe können in regelmäßigen Reports durch das DIFA dargestellt und den teilnehmenden Ärzten zur Verfügung gestellt werden.

Der Weg zur Teilnahme ist für alle Ärzte komfortabel: Nach Installation einer entsprechenden Software per Fernwartung mittels TeamViewer in der Praxis ist kein aktives oder den Praxisablauf beeinträchtigendes Zutun des Arztes mehr nötig.

## Datenhoheit und Datensicherheit

Die Daten aus dem AIS werden extrahiert, anonymisiert und datenschutzrechtlich abgesichert zur Verfügung gestellt. Somit bilden diese Daten in gewissem Sinne die tägliche ärztliche Dokumentation ab. Alle personenbezogenen Daten wie Name, Adresse, Geburtsdatum werden anonymisiert. Aus datenschutzrechtlichen Gründen können dabei Freitexte und PDF-Dateien noch nicht berücksichtigt werden. Auch eine Übernahme von Daten, die dem Gendiagnostikgesetz unterliegen, erfolgt nicht. Die Daten lagern zudem auf einem sicheren Server beim DIFA, welcher in Deutschland steht und so deutschen datenschutzrechtlichen Standards genügt. Sie werden nicht an Dritte weitergegeben. Bereits im Vorfeld wurde das Projekt durch unabhängige Gutachter hinsichtlich Daten- und Persönlichkeitsschutz geprüft und erfüllt in jeder Hinsicht höchste Ansprüche, die an die Risikovorsorge gestellt werden. Die Anonymisierung führt darüber hinaus dazu, dass die in der Datenschutzgrundverordnung (DSGVO) niedergelegten Regeln nicht tangiert werden.

## Methodik der Datenauswertung

Gegenwärtig beteiligten sich bereits 105 niedergelassene Urologen an UROscience mit der Bereitstellung von Daten. Dadurch liegen für insgesamt 2,3 Mio. Patienten anonyme Versorgungsdaten vor. Die Daten reflektieren das Versorgungsgeschehen seit dem 01.01.2011. Die Daten werden über die oben beschriebenen Aktivitäten zur Sicherung der Anonymität hinaus nicht vorverarbeitet oder kuratiert. Um das Potenzial und die Repräsentativität der vorliegenden Daten zu demonstrieren, wurde eine Auswahl deskriptiver Basisanalysen durchgeführt.

Diese können sich auf verschiedene Parameter und Fragestellungen beziehen. Es können z. B. Fallanalysen durchgeführt werden. Diagnose‑, Behandlungs‑, Verordnungs- oder Abrechnungsfälle können für einen definierten Zeitraum als Zählwerte dargestellt werden, wobei nicht erfasst wird, ob individuelle Patienten einmal oder mehrmals pro Zeiteinheit betroffen waren. Veränderungen in der Dynamik derartiger Zählwerte können über die Zeit beobachtet werden und so zu aufschlussreichen Erkenntnissen führen.

Die Dateninhalte können auch als individuelle Diagnose- und Therapieverläufe „longitudinal“ über die Zeit dargestellt werden. Daraus lässt sich beispielsweise ableiten, welche Abläufe in Zusammenhang mit einer Diagnose (oder Kodiagnosen) stehen, wie oft ärztliche Leistungen in Anspruch genommen werden und wie sich in der Folge bestimmte Laborwerte als Indikatoren für den Schweregrad einer Erkrankung verändern. Vor dem Hintergrund beispielsweise von Analysen zur aktiven Überwachung beim Prostatakarzinom wäre das ebenso interessant wie zur Darstellung von Verläufen bei Harnwegsinfekten. Auch könnte man sich Therapieinterventionen im Zusammenhang mit der BPH vorstellen.

Auch der zeitliche Verlauf von Verordnungen sowie Therapiewechsel können nachvollzogen werden. Durch Hinzunahme der Abrechnungsziffern können orientierende Kostenschätzungen, soweit sie in der betreffenden Praxis angefallen sind, vorgenommen werden. Einschränkend sollte in diesem Zusammenhang erwähnt werden, dass einem Individuum nur die Versorgungsdaten der datenspendenden Praxis zugeordnet werden können.

Einzelne Dateninhalte können zueinander in Beziehung gesetzt und über den Zeitverlauf nachvollzogen werden. Ist beispielsweise das Datum der Erstdiagnose einer chronischen Erkrankung bekannt, kann die Zeit bis zum Auftreten von Komplikationen oder einer Verschlechterung der Laborindikatoren mit bestimmten Therapien oder anderen Interventionen in Beziehung gesetzt werden. Daraus ergibt sich eine Vielzahl von analytischen Perspektiven.

## Ergebnisse

**Figure 2:**
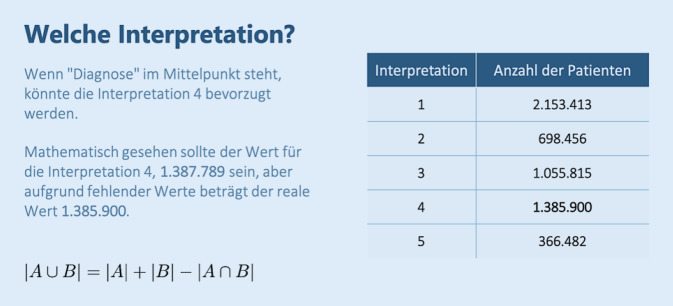

Um das Potenzial und die Repräsentativität eines Datensatzes zu demonstrieren wird nicht selten die reine Zahl, gerne auch als „*n* = x“, bemüht. Diese Kennzahl soll plakativ auch die Aussagekraft gemachter Schlussfolgerungen unterstreichen – kann aber trügerisch Sicherheit vermitteln, wie der folgende Datenauszug aus der Rohdatensammlung eindrücklich belegt: Hierzu wurden verschiedene Interpretationsansätze der Frage nach der reinen Zahl der Patientendatensätze in der Sammlung zu einem Analysekatalog zusammengestellt und getrennt ausgewertet (Abb. 2):

1. Gesamtzahl der Patienten in der Datenbank, unabhängig davon, ob wir Diagnose‑, Therapie- oder Laborwerte für sie haben oder nicht (es gibt Patienten in der Datenbank ohne die genannten Informationen),
2. Gesamtzahl der Patienten, für die wir „Dauerdiagnosen“ haben,
3. Gesamtzahl der Patienten, für die wir „Akutdiagnosen“ haben,
4. Gesamtzahl der Patienten, für die wir die „Diagnose“ (Dauerdiagnosen ODER Akutdiagnosen) haben,
5. Gesamtzahl der Patienten, für die wir die „Diagnose“ (Dauerdiagnosen UND Akutdiagnosen) haben.

### Top 10 der Diagnosen

Die Frage „Was machen Urologen in der Praxis?“ führt unweigerlich zur Frage nach den 10 häufigsten Diagnosen. Auch hier gibt es einen „Analysespielraum“ und unterschiedliche Interpretationen der Frage sind möglich:

1. häufigste Diagnosen basierend auf Akutdiagnosen,
2. häufigste Diagnosen basierend auf Dauerdiagnosen,
3. häufigste Diagnosen (Vereinigung von Akutdiagnosen und Dauerdiagnosen).

**Figure 3:**
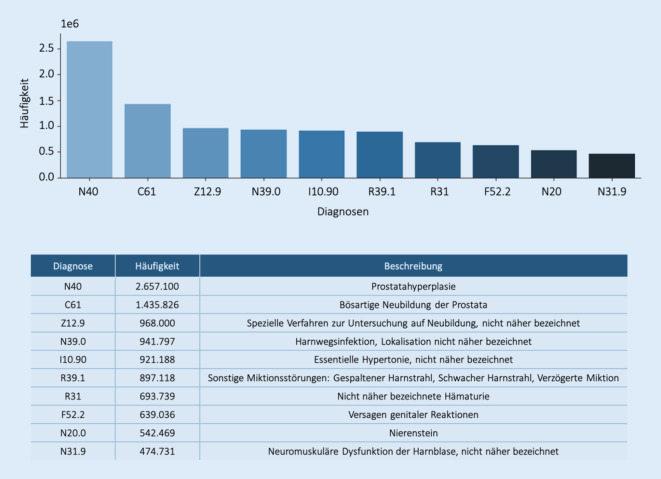

In der Auswertung auf Abb. 3 wird nur das Auftreten eines Diagnoseereignisses („Akutdiagnose“) gezählt und dabei die Tatsache ignoriert, dass ein Patient mehrmals dieselbe Diagnose haben kann.

**Figure 4:**
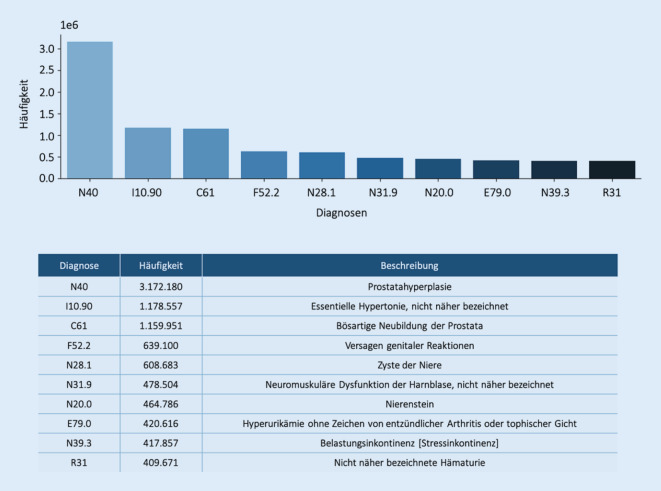

In Abb. 4 wird nur die Häufigkeit der Dauerdiagnosen oder mehrmalig erfassten Diagnosen dargestellt. 7 der 10 genannten Dauerdiagnosen werden auch unter den Top 10 der Akutdiagnosen gefunden. Die Prostatahyperplasie wurde in beiden Kategorien am häufigsten festgestellt; die bösartigen Neubildungen der Prostata befinden sich auf Platz 2 bzw. 3. Dies erklärt sich durch die vergleichsweise hohe Prävalenz der Erkrankungen. Durch die Tatsache, dass Patienten im Verlauf der in der Datenbank erfassten Jahre zu unterschiedlichen Zeitpunkten (zum ersten Mal) die Praxen aufsuchen, ist die Anzahl der Akutdiagnosen zur Prostatahyperplasie relativ hoch, obwohl es sich dabei um eine chronische Erkrankung handelt. Die Hintergründe zu diesen Beobachtungen ließen sich durch weitere Analysen auf der Ebene einzelner Patienten und deren Krankheitsverlauf klären.

Sowohl bei den Akut- als auch bei den Dauerdiagnosen fällt die Nennung der essentiellen Hypertonie auf. Dies ist der Tatsache geschuldet, dass man in der Praxis (wie sonst auch) angehalten ist, möglichst vollständig zu Kodieren. Hintergrund ist, dass die Kodierqualität eine Rolle bei den Ermittlungen der Morbidität und damit des morbiditätsorientierten Gesamtvolumens (MGV), sprich des Gesamthonorarbudgets der jeweiligen Kassenärztlichen Vereinigung, eine entscheidende Rolle spielt. Deshalb werden häufig anamnestische Nebendiagnosen als Dauerdiagnosen mit abgespeichert. Da diese Nebendiagnosen sehr häufig sind, „überlagern“ sie möglicherweise deshalb in den Top 10 die eigentlichen urologischen Behandlungsdiagnosen. Auf der anderen Seite ist dieses Phänomen eine typische Beobachtung in vielen Fachgruppen wegen der hohen Prävalenz und der damit verbundenen Folgeerkrankungen und (Ko‑)Verordnungen.

### Altersverteilung und Geschlecht der Patienten „in der Praxis“

Ein weiterer Schwerpunkt der Basisanalyse bezog sich auf die Altersverteilung der Patienten, die durch niedergelassene Urologen versorgt werden. Auch diese Frage kann auf Basis der Datenstruktur unterschiedlich interpretiert werden:

1. Altersstruktur der Patienten (mit oder ohne Diagnose),
2. Altersstruktur der Patienten mit mindestens einer Diagnose (zeitaufwändige Analyse).

**Figure 5:**
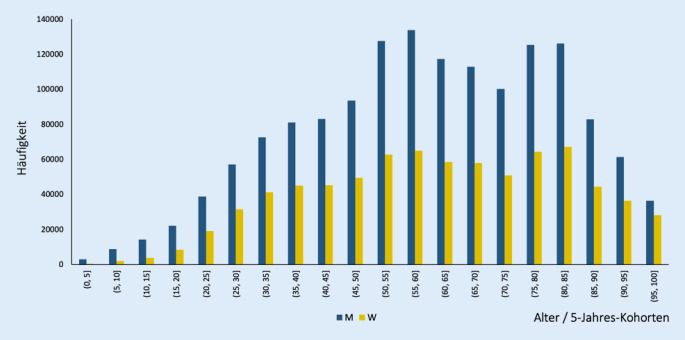

Weitere Interpretationen wären möglich. Abb. 5 stellt den ersten Fall dar.

In Abb. 5 ist die Altersverteilung der Patienten in Form eines Histogramms dargestellt. Die Balken repräsentieren jeweils 5‑Jahres-Kohorten. Der Mittelwert des Lebensalters liegt bei ca. 60 Jahren. Insgesamt ergibt sich der Eindruck einer Normalverteilung. Zwei bedeutsame Schlussfolgerungen können aus der Analyse gezogen werden:

#### Niedergelassene Urologen versorgen alle Altersgruppen

Während die Prävalenz der Top-10-Diagnosen in fortgeschrittenem Alter höher ist, können wir trotzdem eine signifikante Anzahl junger Patienten beobachten. Insgesamt finden sich ca. 64.000 Patientinnen und Patienten in der Altersgruppe 0–20 Jahre.

#### Niedergelassene Urologen versorgen beide Geschlechter

Der Anteil der Männer sinkt mit zunehmendem Alter ab. Er beträgt 87 % bei 0‑ bis 5‑jährigen, 76 % bei > 5- bis 20-jährigen und 65 % bei > 40- bis 90-jährigen Patienten. Der nochmals sinkende Anteil der Männer bei > 90-Jährigen lässt sich vermutlich auf die höhere Lebenserwartung der Frauen zurückführen.

### Behandlungszeitraum in der Praxis

Patienten werden über unterschiedliche Zeiträume in der urologischen Praxis behandelt. Auch hier gibt es unterschiedliche Perspektiven und Darstellungsmöglichkeiten:

1. Zeitdauer als Patient/-in in der Praxis basierend auf Diagnosen,
2. Zeitdauer als Patient/-in in der Praxis basierend auf Therapien,
3. Zeitdauer als Patient/-in in der Praxis basierend auf Labordaten,
4. Zeitdauer als Patient/-in in der Praxis basierend auf allen verfügbaren Daten (komplexe Analyse).

Die Analyse bezieht sich auf den ersten Fall.

**Figure 6:**
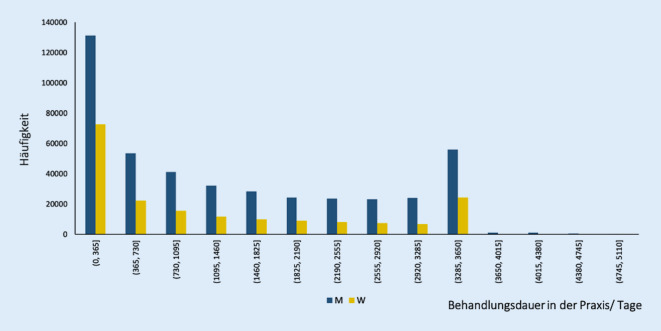

Bei einem Vergleich der Behandlungszeiträume von einem bis zu 10 Jahren (basierend auf Diagnosen) ist der relativ größte Anteil der Patienten einem Behandlungszeitraum von bis zu einem Jahr zuzuordnen (32 %). Über eine zunehmende Anzahl von Jahren nimmt der Anteil der Patienten bis zum Zeitraum von 8 bis 9 Jahren kontinuierlich ab. Danach ist ein starker Anstieg bei den Patienten mit einer Behandlungsdauer von 9 bis 10 Jahren zu beobachten. Diese Gruppe stellt die zweitgrößte Kohorte (13 %) in Bezug auf Behandlungsdauer in der Praxis dar. In weitergehenden Analysen könnten die zugrunde liegenden Diagnosen, Altersgruppen oder Therapien untersucht werden. Es darf vermutet werden, dass z. B. die Prostatahyperplasie besonders häufig bei den Patienten mit langer Behandlungsdauer vorkommt (Abb. 6).

Auch bei der Analyse der Behandlungsdauer wird deutlich, dass es keine unterschiedliche Dynamik bei den Geschlechtern gibt – der Anteil von Männern und Frauen verändert sich nicht.

## Diskussion und Zusammenfassung

Bei den vorgestellten Auswertungen wurden klassische analytische Verfahren eingesetzt, um erste Erkenntnisse zum Charakter der UROscience-Datenbank zu gewinnen. Der „ungefilterte“ Blick auf die Daten und der deskriptive Charakter der Ergebnisse belegt dabei klar, dass bei jeder Analyse die Struktur der vorliegenden Daten berücksichtigt werden muss. Vordergründig einfache Fragen erfordern somit präzise Definitionen des analytischen Vorgehens, und die Ergebnisse müssen darauf bezugnehmend interpretiert werden. Dafür ist ein inhaltlicher intensiver Austausch zwischen den mit der Datenbank vertrauten Analysten, den Praxisbetreibern und den auf der Datenbank Forschenden erforderlich. Nur so kann der Auswertealgorithmus kontinuierlich verbessert werden und die Güte und Gültigkeit der Analysen gesteigert werden.

**Figure 7:**
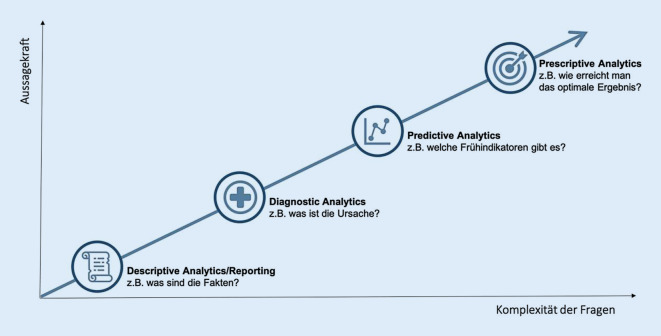

Schon jetzt wecken die vorliegenden deskriptiven Ergebnisse Interesse an tiefergehenden Analysen. Dafür können moderne analytische Methoden herangezogen werden, die man unter dem Begriff „advanced analytics“ zusammenfasst ([18]; s. Abb. 7). Es werden dafür u. a. Modelle neuronaler Netzwerke eingesetzt, weshalb der Begriff „künstliche Intelligenz“ verwendet wird. Mit ihrer Hilfe können deskriptive um prädiktive Analysen ergänzt werden, wobei durch die Erkennung von Mustern und Gesetzmäßigkeiten in den Daten Ergebnisse vorausgesagt werden können.

So könnte z. B. die Hypothese entwickelt werden, welche Patienten von einer Therapie besonders profitieren und wie hoch die Wahrscheinlichkeit ist, dass dies tatsächlich passiert. Die präskriptive Analytik geht noch einen Schritt weiter und empfiehlt Maßnahmen zur Optimierung. Derartige Analysen profitieren von reichhaltigen („tiefen“) Daten und großen Datenbeständen, weshalb der weitere Ausbau von UROscience angestrebt wird.

In der Zusammenfassung wir deutlich, dass neben der Implementierung der automatisierten Krebsregistermeldung im UROgister auch über die Extraktion von Daten aus dem AIS in UROscience ein Datenpool entstehen kann, der für viele Fragestellungen zur Versorgungsrealität – auch berufspolitisch – genutzt werden kann. Und auch, wenn die Erkenntnis, dass das Fachgebiet der Urologie mehr ist als „Alters- oder Männerheilkunde“, längst überholt ist, kann nun mit Zahlen eindrücklicher belegt werden, was in der urologischen „Praxis“ passiert. Mit dem zunehmenden Ausbau dieser Struktur wird UROscience auch wichtige Basis für den Dialog mit Kostenträgern und Politik sein, wenn hier aus „eigener Hand“ Daten und Fakten vorgelegt werden können. Auch wäre eine Einbindung in andere Projekte, wie etwa im Rahmen der Beteiligung als Konsortialpartner im Innovationsfondsprojekt „Stand und Weiterentwicklung der ambulanten spezialfachärztlichen Versorgung im Bereich ‚Urologische Tumore‘ (ASV-WE)“, das auf Initiative von Prof. Lothar Weißbach und unter Federführung des aQua-Instituts im April 2021 starten wird, wünschenswert.

Darüber hinaus können auch aus anderen Bereichen Fragestellungen eingereicht werden. Ansprechpartner ist hierbei das Deutsche Institut für Fachärztliche Versorgungsforschung GmbH (DIFA): https://difa-vf.de/impressum, info@difa-vf.de.

## Fazit

Auf Basis der bereits heute vorhandenen Daten steht uns eine vielseitig einsetzbare Stichprobe für die Darstellung des Versorgungsalltags aus der urologischen Praxis zur Verfügung. Selbst wenn ein signifikanter Anteil der Datensätze wegen Unvollständigkeit oder Inkonsistenz unberücksichtigt bleiben müsste, wäre zu erwarten, dass viele Fragestellungen zur Versorgung bei niedergelassenen Urologen und zur Epidemiologie untersucht werden könnten. Alle Urologen sind aufgerufen, sich hieran zu beteiligen: mit Daten und/oder mit Fragestellungen.
